# Polish Adaptation and Psychometric Validation of the METEO-Q in Healthy, Cardiac, and Psychiatric Samples

**DOI:** 10.3390/jcm15082853

**Published:** 2026-04-09

**Authors:** Krystian Konieczny, Karol Karasiewicz, Karolina Rachubińska, Krzysztof Wietrzyński, Marianna Mazza, Monika Mak

**Affiliations:** 1 Department of Clinical Psychology and Psychoprophylaxis, Institute of Psychology, University of Szczecin, 71-017 Szczecin, Poland; 2 Department Social and Developmental Psychology, Institute of Psychology, University of Szczecin, 71-017 Szczecin, Poland; 3 Department of Health Psychology, Pomeranian Medical University in Szczecin, 71-460 Szczecin, Poland; 4 Unit of Psychiatry, Fondazione Policlinico Universitario Agostino Gemelli IRCCS, 00168 Rome, Italy; 5 Department of Neurosciences, Università Cattolica del Sacro Cuore, 00168 Rome, Italy

**Keywords:** meteoropathy, meteosensitivity, weather, psychometric validation, clinical psychology

## Abstract

**Background**: Although the concepts of meteoropathy and meteosensitivity are not included in official classifications, such as the ICD-11 or DSM-5, they are increasingly being studied as potential symptom complexes linking weather variability to health status. The METEO-Q questionnaire, originally developed in Italy, has been adapted in Japan and Turkey, where it has demonstrated satisfactory reliability parameters, although the authors emphasized the need for further verification of the tool’s temporal stability. The present study aimed to adapt METEO-Q to the Polish language and conduct a critical assessment of its factor structure, measurement invariance, and validity in clinical groups. **Methods**: This cross-sectional study involved 1128 adults: healthy individuals (*n* = 711), cardiac outpatients (*n* = 194), and subclinical group with diagnosed mental disorders (*n* = 223). Data from healthy participants were divided into a training sample (*n* = 426) for exploratory factor analysis (EFA) and a test sample (*n* = 285) for confirmatory factor analysis (CFA). Measurement invariance was assessed in the clinical groups. Validity was verified through correlations with a list of 21 symptoms and measures of anxiety and worry about climate change. **Results**: A two-factor model (meteoropathy and meteosensitivity) was better fitted to the data than a one-factor model, which is consistent with findings from Italian, Japanese, and Turkish studies. However, absolute fit indices in the test sample indicated significant model misfit [CFA: χ^2^ (43) = 210.192, *p* < 0.001, RMSEA = 0.120, CFI = 0.927], suggesting the presence of local errors in the tool’s structure. The reliability of the subscales was high (α from 0.86 to 0.93). Multi-group analyses suggested metric and scalar invariance. Patients with mental disorders obtained the highest scores, while cardiac outpatients reported a lower level of meteoropathy (M = 6.13) than healthy individuals (M = 7.24). **Conclusions**: METEO-Q demonstrates a stable two-factor structure and high internal consistency. The obtained RMSEA index (0.12), although indicative of some misfit, is similar to results obtained in other adaptations, such as the Japanese (RMSEA = 0.10) and the Turkish (RMSEA = 0.11), which suggests it is a consistent feature of this tool across different cultural contexts. Accordingly, the instrument is suitable for research purposes; however, its clinical application requires considerable caution and further work to optimize the model.

## 1. Introduction

In clinical observations, some patients have reported that the deterioration in their physical or mental condition coincides with specific weather patterns or atmospheric phenomena, including precipitation, strong winds, and barometric pressure changes. Although such associations are often regarded as subjective interpretations, empirical findings from medical biometeorology indicate measurable links between meteorological variability and human health outcomes. Meteoropathy and meteosensitivity, while not recognized in ICD-10, ICD-11, or DSM-5, are increasingly described as potential syndromes or emerging nosological categories [1].

The etymology of meteoropathy comes from the Greek words “meteora” (things high in the air or celestial phenomena) and “pathos” (illness, suffering, and pain) [1]. People identified as “meteoropathic” tend to develop new illnesses or symptoms or see a worsening of their current health issues as a result of climatic or weather shifts. On the other hand, those known as “meteorosensitive” are biologically predisposed to experience the effects of specific weather events on both their mental and physical states.

Research results indicate the existence of links between weather/atmospheric variables and health. Evidence for this is provided by research at the intersection of biometeorology and medicine (medical biometeorology) in both somatic and mental health.

Cardiac patients are particularly vulnerable to the effects of meteorological changes, which may constitute a risk factor for deterioration in health or death [2,3], especially in terms of temperature increase and decrease [4,5]. These trends are particularly significant among the senior population; the effects of weather temperature change on all cardiovascular disease (CVD) hospitalizations are greater in older age groups, particularly those aged 75–89 years and ≥90 years, than in those ≤64 years old [6].

Meteorological factors are also important in somatic diseases of various origins, such as pain [7] (including osteoarthritis [8], weather-related pain [9]), asthma [10], hemorrhagic stroke and subarachnoid hemorrhage [11], fibromyalgia [12], multiple sclerosis [13,14], eating disorders [15], obesity [16], and others. It should be noted that the compounds described and the results of the studies vary depending on the methodology used and the groups studied, and some results contradict each other, suggesting the possibility of other uncontrolled variables underlying the mechanisms described and the symptoms observed.

Correlations between meteorological variables and seasonality have also been observed in mental health. Data from Italy suggest a strong positive correlation between average monthly temperatures and admissions to an acute psychiatric inpatient unit in Italy. This relationship is particularly pronounced in patients diagnosed with bipolar disorder (BD) [17]. Other studies have suggested a direct link between meteoropathy and suicide attempts among euthymic patients with bipolar disorder [18]. There is also a consistent correlation between rising temperatures and increased suicide rates in the literature.

Data from Hefei (China) indicated a significant relationship between ambient temperature and hospital admissions for schizophrenia. Higher temperatures were associated with an increased risk of hospitalization for this condition, with a delayed effect. Specifically, a temperature of 28 °C over a 0–4 day lag period correlated with a 7% increase in schizophrenia-related admissions [19].

In addition, research on climatic and seasonal factors in Bosnia and Herzegovina indicates the existence of several correlations. For mood disorders in 2010, most patients were admitted in spring (28%), followed by winter (27%), fall (25%), and summer (20%). In 2011, the highest admission rate was in winter (28%), followed by autumn and spring (26%) and summer (20%). This study found a strong, statistically significant correlation between mood disorders and temperature, indicating that an increase in temperature affects the number of hospitalizations with this diagnosis. For schizophrenia and related disorders, the study found an increased number of patients hospitalized during the summer months. There was a strong statistically significant correlation with temperature, suggesting that higher temperatures led to more frequent hospitalizations in this group of patients. For mental and behavioral disorders due to psychoactive substance use, the highest admission rate was in fall (42%), whereas in 2011, it was in spring (30%) [20].

High temperatures and heat waves are the most frequently analyzed exposures, showing a correlation with an increase in the incidence of major depressive disorder (MDD) and the severity of anxiety symptoms. A study conducted on the Taiwanese population showed that in regions with average annual temperatures above 23 °C, each additional 1 °C increase was associated with a 7% increase in the incidence of MDD [21]. Simultaneously, the United States has seen an increase in the frequency of crisis reports (e.g., to helplines) among young adults during periods of elevated air temperatures [22]. Collectively, these findings support the clinical relevance of weather-related symptoms and provide a rationale for their systematic assessment using standardized psychometric tools.

Taken together, these data suggest that individual sensitivity to ordinary weather variations is clinically meaningful and should be quantified reproducibly. In this context, Mazza et al. [23] proposed METEO-Q, a self-report questionnaire developed to detect meteoropathy and meteorosensitivity and described by the authors as the first questionnaire aimed at measuring symptoms of meteoropathy. The instrument was formulated to assess sensitivity to climate changes and their impact on symptomatologic modifications, and it consists of 11 items and a structured symptom checklist.

Meteoropathy (items 1–5) refers to the emergence of symptoms or exacerbation of pre-existing complaints due to changing atmospheric conditions.

Meteosensitivity (items 6–11) refers to biological susceptibility to changes in meteorological conditions, expressed both somatically and psychologically. In the present study, this should be understood as self-reported perceived susceptibility rather than objectively measured biological reactivity.

The checklist evaluates 21 physical and psychological symptoms most frequently associated with meteorological variability (e.g., asthenia, irritability, headaches, and sleep or appetite disturbances). All responses were rated on a 5-point Likert scale, from 0 (absent) to 4 (severe). Importantly, this construct has already been examined across different cultural contexts. The Japanese [24] adaptation confirmed a two-factor solution consistent with earlier work and showed high internal consistency for both factors, whereas the Turkish [25] adaptation also supported a two-factor structure, accounting for 68.59% of the variance, and demonstrated acceptable final CFA fit (initial model RMSEA = 0.11, final modified model RMSEA = 0.08, CFI = 0.98, SRMR = 0.04), together with good test–retest reliability. These findings suggest that the instrument captures a relatively stable and reproducible distinction between meteoropathy and meteosensitivity across cultural settings. Although further validation work remains warranted. A Polish adaptation has also been reported by Oniszczenko; however, it has never been published with its psychometric characteristics [26].

The purpose of this study was to adapt the METEO-Q for the Polish population. The adaptation process was carried out in multiple stages involving healthy Polish adults, as well as cardiac outpatients and individuals with subclinical psychiatric disorders. This design was adopted because the original METEO-Q was developed primarily in healthy participants, whereas both the broader literature and earlier clinical observations suggest that weather-related symptom sensitivity may also be relevant in populations with somatic and psychiatric burdens. Specifically, the analyses were conducted in three steps: (1) exploratory analysis in a subgroup of healthy adults (*n* = 426, 59.9% of the healthy adult sample), (2) confirmatory analysis in a subgroup of healthy adults (*n* = 285, 40.1% of the healthy adult sample), and (3) measurement invariance analysis in a sample of cardiac outpatients and individuals with subclinical psychiatric disorders (*n* = 417).

## 2. Materials and Methods

### 2.1. Study Procedure

In the first step, approval was obtained from Marianna Mazza to adapt the METEO-Q to Polish conditions. Next, approval was obtained from the Bioethics Committee at the Institute of Psychology at the University of Szczecin.

In the next step, three independent translators (native speakers) involved in translating scientific texts translated the original Italian version of the METEO-Q. These versions were evaluated by three competent judges for content accuracy and linguistic correctness. Next, a pilot study was conducted to test the clarity of each item and the general construct. The participants were psychologists working in the clinical field, two of whom were scientists. Their task was to answer three questions: (1) How would you personally respond to each item, (2) How do you assess the linguistic accuracy of the entry? And (3) To what extent does the entry correspond to the concept of meteoropathy or meteosensitivity? Based on the suggestions of competent judges, the wording of one of the original test items was changed. The judges pointed out a potential misunderstanding in the Polish population regarding the symptom described as “asthenia” in the symptom list. Accordingly, the wording of the item was changed, as suggested by the judges, to “chronic fatigue” (Pol. “przewlekłe zmęczenie”), representing the most closely related wording.

As a result of this procedure, the best translation was selected separately for each test item, and a version of the sheet was created, which was used in the next stages of the survey. The survey was conducted in accordance with the ethical principles of the Declaration of Helsinki. Each participant provided written consent to participate in the study and had the opportunity to ask questions and obtain answers from the psychologist/researcher conducting the study.

The collected data were subjected to statistical analysis to determine the psychometric properties of the METEO-Q in its Polish adaptation.

### 2.2. Participants

The study was conducted among a total of *n* = 1128 participants (*n* = 417 in both clinical groups, 37% of the study sample; *n* = 711 in the healthy adult group, 63% of the study sample). The study included *n* = 713 women (63.2% of the study sample), *n* = 415 men (36.8% of the study sample). The participants were between 18 and 92 years old (M = 42.9 years; SD = 18.3; MD = 43 years). A detailed description of the study sample and its characteristics is presented in Table 1.

In the sample of healthy adults, two subgroups were identified: the Train sample [*n* = 426, 59.9%)] and the Test sample [*n* = 285, 40.1%)]. In the following stages, the data in the Train sample were used to estimate the psychometric properties of the METEO-Q scale in exploratory analyses, while the data in the Test sample were used to verify the goodness of fit of the models in confirmatory analyses. The results of the comparison in Table 2 support the assumption that the Train and Test samples do not differ in terms of age, gender, education, or place of residence; thus, in this sense, they are parallel.

Validation studies were conducted using two methods: purposive selection (sample of cardiac outpatients) and the snowball method (general group and subclinical psychiatric participants). All study groups were surveyed using the paper-and-pencil method. The survey of cardiology outpatients was conducted at health facilities in West Pomeranian Voivodeship, Poland. During the visit to the facility, the doctor prequalified the outpatient to participate in the study, taking into account, among other things, his cognitive ability to fill out the sheet and his diagnosed cardiac disease. The subclinical group of psychiatric participants was obtained using the snowball method based on the reports of the study participants. Participants who were in closed psychiatric institutions or psychiatric day wards were not included. The condition of the participants in this group was stable. Participants were assigned to groups based on their statement of mental health and past psychiatric treatment. The subclinical sample of psychiatric participants included those who declared psychiatric treatment but simultaneously declared no cardiac treatment. Also included in this group were those declaring drug or alcohol use and receiving psychiatric treatment; however, those (declaring) alcohol or drug use who did not receive psychiatric treatment were not included.

The sample of healthy adults were selected using the snowball method. Individuals who declared drug use, psychiatric treatment, psychotherapy, cardiac diseases (e.g., myocardial infarction, congenital heart defects, implanted by-passes, implanted defibrillator, past cardiac surgery, hypertension, stroke), those under 18 years of age, and dependents were excluded from the group.

In the process of analyzing the collected data, the data of those who filled out the questionnaires unreliably, that is, their answers were extremely different from the others, were excluded from further analysis based on the Mahalanobis distance method. As a result, 102 observations (8.9% of all surveyed individuals) were excluded from the analyses (χ^2^ > 4.21; df = 11; *p* < 0.01)

### 2.3. Measures

This study used the following tools to determine the psychometric properties of the METEO-Q:

#### 2.3.1. Meteoropathy Questionnaire (METEO-Q)—Experimental Polish Version

The METEO-Q is a self-report instrument developed by Mazza et al. [23] to assess meteoropathy and meteosensitivity. It consists of 11 items and a checklist of 21 physical and psychological symptoms associated with meteorological changes. Responses are provided on a 5-point Likert scale ranging from 0 (“absent”) to 4 (“severe”). Items 1–5 measure meteoropathy, and items 6–11 measure meteosensitivity. The symptom checklist covers 21 disturbances frequently associated with meteorological variability (e.g., asthenia, irritability, headaches, concentration difficulties, insomnia, and appetite changes). In the original validation, the questionnaire demonstrated satisfactory factorial validity and internal reliability (Cronbach’s α = 0.81 for items 1–5; α = 0.87 for items 6–11). An experimental Polish version was prepared for the present study. All items were translated from Italian into Polish by translators (native speakers) and subsequently reviewed by clinical psychologists for content accuracy and for linguistic clarity.

#### 2.3.2. Climate Anxiety Scale (CAS)—Polish Version

The CAS was originally developed to measure symptoms of climate anxiety [27]. The Polish adaptation [28] consists of 13 items rated on a 5-point Likert scale (1 = never to 5 = almost always). Previous studies confirmed high internal consistency in the Polish version (Cronbach’s α between 0.87 and 0.92). Measurement invariance was supported across sex, age, and education.

#### 2.3.3. Climate Change Worry Scale (CCWS)—Polish Version

The CCWS is a 10-item measure designed to assess recurrent worry related to climate change [29]. Responses are provided on a 5-point Likert scale (1 = never to 5 = always), yielding total scores ranging from 10 to 50. Higher scores indicate a greater intensity of climate change–related worry. The Polish adaptation [30] demonstrated excellent reliability (Cronbach’s α = 0.93; McDonald’s ω = 0.93) and good model fit for a unidimensional structure (CFI = 0.997, TLI = 0.996, RMSEA = 0.043). Test–retest reliability was acceptable (ICC = 0.66). Convergent validity was supported by correlations with pro-environmental behaviors, subjective experience of climate change, anxiety, and depression.

#### 2.3.4. Demographic Survey

An author-designed questionnaire was administered to collect basic demographic data, including sex, age, education level, and place of residence.

### 2.4. Statistical Analyses

In the Healthy Adults sample, an exploratory factor analysis (EFA) was conducted based on a random subset of 60% of respondents (the so-called “learning sample”) freely fitting a one- and two-factor structure to describe meteoropathy and meteosensitivity in the population of healthy Polish adults. Subsequently, in the Healthy Adults sample, confirmatory factor analyses (CFA) were conducted in a random subset comprising 40% of healthy adults (the so-called “Test Sample”), independent of the learning sample, to test the fit of the one-factor and two-factor structures describing meteoropathy and meteosensitivity in healthy Polish adults. In the same test sample, confirmatory factor analyses were also conducted to test the fit of the univariate and bivariate models (theoretical and empirical, that is, estimated in the learning sample) and to assess the goodness of fit of these models in describing the studied construct in healthy Polish adults. Next, a measurement invariance analysis was conducted for the model selected in the previous step in the population of cardiac outpatient and psychiatric subclinical samples. In the final step, convergent and differential validity were assessed by correlating the METEO-Q scale measurement results with the Climate Anxiety Scale (CAS) and Climate Change Worries Scale (CCWS) measurement results in a sample of healthy adult outpatients and subclinical group (regardless of health status).

## 3. Results

### 3.1. Reliability

To estimate the reliability of the METEO-Q, a unidimensional model (total score) and a two-dimensional model were subjected to internal consistency analysis using Cronbach’s α [31] separately in the Train [*n* = 426] and Test [*n* = 285] subsamples of Healthy Adults. We considered α > 0.70 to be an indicator of high reliability and Average r > 0.25 to be indicative of satisfactory internal consistency among the scale (Table 3).

The analysis results revealed high reliability for both the total score and the two subscales, as indicated by Cronbach’s α of >0.80. Furthermore, it can be assumed that the measurement reliability in the healthy adults population is likely to be higher than α = 0.70. In addition to reliability indices, descriptive statistics were calculated for meteoropathy and meteosensitivity scores across the three study groups (healthy adults, cardiological outpatients, and subclinical psychiatric participants). Table 4 presents the minimum and maximum values, means and standard deviations, medians with median absolute deviation, skewness, kurtosis, and Shapiro–Wilk test values. These indices provide an overview of the distribution of scores and allow for a comparison of the central tendency and variability between clinical and non-clinical samples.

### 3.2. Factorial Validity

To verify the factorial validity of the METEO-Q, we conducted an exploratory factor analysis (EFA) using maximum likelihood (ML) estimation (assuming that normality of METEO-Q scores is met due to Shapiro–Wilk W > 0.9 and Skewness and Kurtosis in the range between −1 and 1) and Oblimin factor rotation (assuming after Mazza et al., 2012 [23] moderate or high inter-factor correlation) in the Train subsample of healthy adults [*n* = 426]. We tested the hypothesis that a two-factor model demonstrates a significantly better fit to the data compared to a one-factor model, and that the structure of the extracted factors aligns with the original theoretical assumptions of the scale developers. We analyzed the Root Mean Square Error of Approximation (RMSEA), Comparative Fit Index (CFI), and Bayesian Information Criterion (BIC) as indices of the overall model fit. We considered RMSEA values < 0.05 and CFI values > 0.95 to indicate excellent model fit; RMSEA values < 0.08 and CFI values > 0.90 to indicate acceptable fit; and RMSEA values > 0.10 and CFI values < 0.80 to indicate poor model fit to the data (Table 5).

The analysis results revealed a statistically significant difference in overall model fit [χ^2^(10) = 81.896, *p* < 0.001], where the RMSEA value of 0.116 for the two-factor model is considerably below 0.13, the lower bound of the 95% confidence interval for the RMSEA of the one-factor model supported by the large change in BIC from 175.0 for the one-factor to 27.8 for the two-factor model. This suggests that the two-factor model demonstrates a significantly better fit to the data in the healthy adult sample compared to the one-factor model, although the overall fit remains only acceptable (CFI < 0.95) and, according to common standards [31], poor, which stems from the relatively small number of parameters used. Nevertheless, the EFA results obtained in the present study were similar to those reported in the Turkish and Japanese adaptations. Specifically, in the Polish population of healthy adults, the two-factor measurement model explained approximately 69% of the variance, compared with 68.6% in the Turkish population and 68.9% in the Japanese population. Moreover, the RMSEA value obtained in this study (0.116) did not substantially differ from the RMSEA reported for the initial two-factor confirmatory model in the Turkish adaptation (0.11) or from that reported in the Japanese adaptation (0.098). It should be noted, however, that the Turkish authors also reported an improved fit for a modified model with three correlated residuals (RMSEA = 0.08).

**Figure 1:**
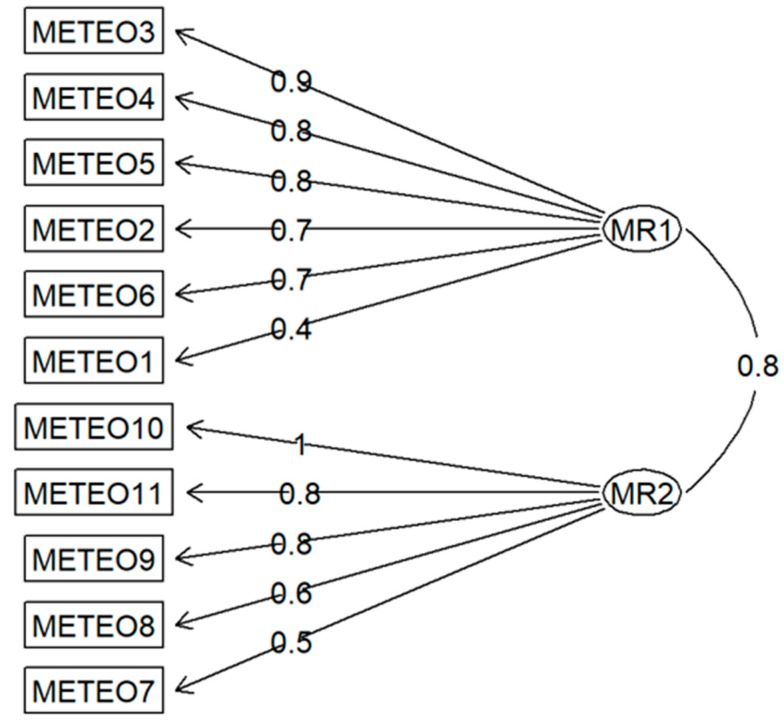
Path diagram of exploratory factor analysis results/EFA model.

As shown in Table 6, the structure of the two factors extracted in the training sample is similar to the original structure assumed by the scale developers, except that Item 6 shows a stronger factor loading on the first factor than on the second factor (contrary to the authors’ assumptions). Moreover, some items exhibited non-trivial (>0.25) factor loadings on both extracted factors, resulting in a non-orthogonal solution and a significant correlation between the identified factors (Figure 1).

### 3.3. Confirmatory Factor Analysis

To validate the factor structure of the METEO-Q, a confirmatory factor analysis (CFA) with maximum estimation (ML) was conducted in the Test sample of healthy adults [*n* = 285]. First, we tested the overall fit of three models: (a) one-factor, (b) two-factor, as assumed theoretically by scale developers, and (c) two-factor, as revealed in previous EFA. As previously mentioned, we analyzed RMSEA, CFI, and BIC as indices of the overall model fit (Table 7).

The analysis results revealed a statistically significant difference in model fit between the one-factor solution and both two-factor solutions [Δχ^2^(1) = 50,618, *p* < 0.001 for the comparison between the one-factor and theoretical two-factor models; Δχ^2^(1) = 36,374, *p* < 0.001 for the comparison between the one-factor and empirically-derived two-factor models]. The difference in fit between the theoretical and empirically-derived two-factor models is negligible, as indicated by differences in BIC, CFI, and RMSEA values below 0.01. Thus, the distinction between these two-factor models is considered trivial. Despite the better fit of the two-factor models compared to the one-factor model, their overall fit to the data can only be considered acceptable [CFI < 0.95, RMSEA > 0.08] (Table 8).

**Figure 2:**
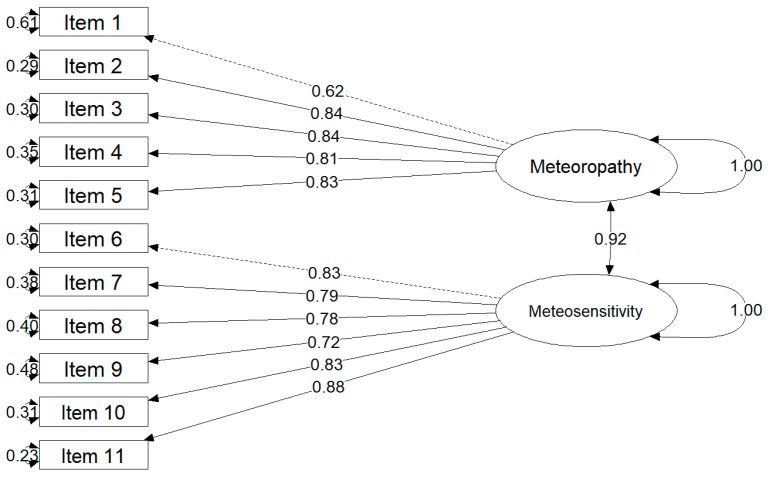
Confirmatory factor analysis path diagram.

Due to the lack of a significant difference in the overall fit between the empirically derived and theoretically assumed two-factor models, we propose using the model theoretically assumed by the scale developers to describe the results of the METEO-Q assessment. The structure of this model estimated in the Test sample of healthy adults is shown in Figure 2.

### 3.4. Measurement Invariance

To verify whether the theoretical two-factor structure is invariant across subclinical populations of cardiological outpatients and subclinical psychiatric group, we conducted a measurement invariance analysis [32]. This procedure tested whether the structure of the METEO-Q was: (a) freely fitted to the data in both the cardiological and psychiatric samples (configural invariance); (b) had equivalent factor loadings across these two populations (metric invariance); (c) had equivalent factor loadings and intercepts across these two populations (scalar invariance); and (d) had equivalent factor loadings, intercepts, and latent variable means across these two populations (latent mean invariance). To test for invariance, we analyzed the chi-square difference (Δχ^2^) and changes in RMSEA and CFI. The results are presented in Table 9.

The analysis results revealed that METEO-Q is strongly invariant in cardiological outpatients and subclinical psychiatric group. Although the χ^2^ difference test revealed that the difference between metric and scalar invariance is significant [*p* < 0.001], the difference is trivial in RMSEA and CFI values (approximately 0.01). This suggests a strong invariance of the METEO-Q measurement across cardiological outpatient and subclinical psychiatric participants.

### 3.5. Convergent and Discriminant Validity

To verify the convergent and discriminant validity of the METEO-Q against weather anxiety and climate change worries, we conducted Heterotrait-Monotrait (HTMT) analysis [33] separately for healthy adult sample (*n* = 711), outpatient cardiological sample and subclinical psychiatric group (*n* = 417). We considered the METEO-Q to demonstrate convergent validity when the Average Variance Extracted (AVE) exceeded the threshold of 0.50. Furthermore, we considered the METEO-Q to demonstrate discriminant validity from other constructs if the maximum shared variance (MSV) between this scale and any other construct was lower than the AVE for the METEO-Q (Table 10).

The results of the analysis revealed that the METEO-Q demonstrated discriminant validity relative to climate anxiety, climate change worries, physical and psychological symptoms associated with meteorological changes across all tested samples. This was evidenced by the Maximum Shared Variance (MSV) being lower than the Average Variance Extracted (AVE) for both meteoropathy and meteosensitivity subscales. Furthermore, neither the meteoropathy nor the meteosensitivity subscales demonstrated convergent validity with these other constructs, as indicated by the Average Shared Variance (ASV) being considerably below 0.50 in all tested samples (Table 11 and Table 12).

### 3.6. Cut-Off Points and Severity Levels

To enable the individual interpretation of METEO-Q results, cut-off points were established for meteoropathy and meteosensitivity in healthy adults, cardiological outpatients, and the subclinical psychiatric group. The cut-offs were determined based on score distributions, following the procedure described by Mazza et al. [23]. Supplementary Table S1 presents the cut-off points defining low, medium, risk, and high severity levels for each group.

### 3.7. Frequencies of Severity Levels

Using these cut-offs, participants were classified into four severity categories. Supplementary Table S2 shows the distribution of individuals across the categories in all three groups of this study. As expected, higher proportions of participants fell into the “risk” and “high” categories in the so called clinical groups, particularly among the psychiatric subclinical group, compared to healthy adults.

## 4. Discussion

This study provides the first systematic psychometric evaluation of the Polish adaptation of the Meteoropathy Questionnaire (METEO-Q). A total of *n* = 1128 adults participated, including healthy adults (*n* = 711), cardiology outpatients recruited in clinical settings with physician involvement (*n* = 194), and psychiatric subclinical participants recruited from the community who reported ongoing psychiatric treatment and met the inclusion/exclusion criteria described in the Methods section (*n* = 223). The instrument was translated, culturally adapted, and assessed for internal consistency, factorial structure, multigroup measurement invariance, convergent and discriminant validity, and preliminary percentile-based severity cut-off points. The present findings suggest that the Polish METEO-Q is internally consistent, structurally coherent, and provides preliminary support for interpretability in research settings. These findings are consistent with evidence that meteorological conditions are linked to biological reactivity, symptom exacerbation, and functional burden in both somatic and psychiatric populations [34,35,36,37,38,39,40,41,42,43,44,45].

Existing studies have shown that atmospheric and meteorological variations are associated with clinically relevant outcomes across multiple systems. Temperature, humidity, barometric pressure, and rapid changes in local weather have been linked to cardiovascular strain and the risk of myocardial infarction, as well as reductions in perceived well-being among patients with cardiovascular diseases [34,39,40,41]. Meteorological shifts have also been associated with headache, fatigue, joint and muscle pain, autonomic complaints, irritability, and mood changes as a symptom cluster that has been described in the biometeorology and clinical medicine literature as “meteoropathy” [23,46]. Fluctuations in ambient temperature and other short-term weather parameters have been associated with increased psychiatric emergency contacts, higher rates of involuntary psychiatric admissions, increased presentations related to schizophrenia and other severe mental disorders, sleep disruption, dysregulated affect, and suicidal behavior [35,36,37,38,42,45,47,48,49]. Severe weather events such as floods and wildfires have also been followed by persistent anxiety, insomnia, depressive symptoms, and post-traumatic stress symptoms that can last for months or years after exposure [43,44]. Together, these data suggest that individual sensitivity to ordinary weather variation may represent a clinically relevant dimension of burden rather than a trivial complaint and warrants a systematic and reproducible assessment [23,43,44].

The first major finding of this study is that the expected two-factor structure of the METEO-Q was supported. The Healthy Adults sample was randomly divided into a Train subsample (*n* = 426) and an independent Test subsample (*n* = 285). In the Train subsample, the EFA showed that a two-factor solution fit better than a one-factor solution. The one-factor model yielded χ^2^ = 149.484 with 44 degrees of freedom (df), RMSEA = 0.146 (95% CI: 0.13–0.16), CFI = 0.885, TLI = 0.857, BIC = 175.02, and explained variance η^2^ = 0.594, respectively. The two-factor model improved all indices: χ^2^ = 67.588 with 34 df, RMSEA = 0.116 (95% CI: 0.10–0.13), CFI = 0.944, TLI = 0.909, BIC = 21.79, and η^2^ = 0.643. This structure was then evaluated in the Test subsample using CFA. Three models were compared: a unidimensional model, a theoretically specified two-factor model, and an empirically modified alternative model. In the Test subsample, the unidimensional model showed χ^2^ = 260.810 with 44 df, RMSEA = 0.135 (95% CI: 0.12–0.15), CFI = 0.906, TLI = 0.882, and BIC = 7156.01. The two-factor model improved fit (χ^2^ = 210.192 with 43 df, RMSEA = 0.120 [0.10–0.14], CFI = 0.927, TLI = 0.907, BIC = 7111.00), and outperformed both the unidimensional and the empirical alternative (χ^2^ = 224.436 with 43 df, RMSEA = 0.125 [0.11–0.14], CFI = 0.921, TLI = 0.899, BIC = 7125.24). The standardized loadings were also high. For the first factor, meteoropathy (items 1–5), the standardized loadings (β) ranged from 0.62 to 0.84. For the second factor, meteosensitivity (items 6–11), the standardized loadings ranged from 0.72 to 0.88. The latent correlation between Meteoropathy and Meteosensitivity was β = 0.92. These data suggest that (i) each set of items defines a coherent latent construct and (ii) the two constructs are strongly associated but not redundant.

In this study, we defined meteoropathy (items 1–5) as the onset or worsening of physical and psychological symptoms temporally associated with changes in the weather or atmospheric conditions. We defined meteosensitivity (items 6–11) as biological susceptibility to meteorological changes, which was expressed as subjective reactivity.

The second major finding concerns internal consistency. Reliability indices were high in both the Train and Test subsamples. In the Train subsample, Cronbach’s α was 0.94 for the total METEO-Q score, 0.86 for meteoropathy, and 0.93 for meteosensitivity. In the Test subsample, Cronbach’s α was again 0.94 for the total score, 0.89 for meteoropathy, and 0.92 for meteosensitivity. The average item–scale correlations (“Average r”) for the two subscales ranged from 0.56 to 0.68. Taken together, these values suggest that both the subscales and the total score show high internal consistency and that the items within each subscale cohere without redundancy. The present results are comparable to or stronger than those reported in the initial Italian validation [23] and subsequent Turkish and Japanese adaptations, which also reported a two-factor structure and satisfactory reliability [24,25].

Third, descriptive comparisons across the examined groups suggest that weather-related complaints may vary between samples. In healthy adults, the mean meteoropathy score (range, 0–20) was 7.24 (SD = 4.61), and the mean meteosensitivity score (range, 0–24) was 7.03 (SD = 5.62). In cardiac outpatients, meteoropathy M = 6.13 (SD = 4.36) and meteosensitivity M = 7.34 (SD = 5.84). In the subclinical psychiatric group, meteoropathy M = 9.58 (SD = 4.26) and meteosensitivity M = 8.97 (SD = 5.58; range, 0–23). Thus, the subclinical sample showed the highest weather-related symptom scores, followed by cardiological outpatients and healthy adult samples. This pattern is compatible with previous observations linking adverse meteorological conditions with psychiatric burden [35,36,37,38,42,43,45,47,48,49]. At the same time, these between-group differences should be interpreted cautiously because the subsamples differed demographically (cardiology outpatients were generally older, whereas subclinical participants were younger and predominantly female), and recruitment strategies differed (in-clinic recruitment with physician involvement for cardiology outpatients vs. community recruitment for subclinical psychiatric patients and healthy adults). Therefore, the present results should not be interpreted as showing that the observed gradient is attributable to clinical status alone, because some portion of it may reflect demographic and recruitment-related differences. Fourth, multigroup measurement invariance analyses provided preliminary support for the approximate comparability of the latent structure of the METEO-Q across cardiological outpatients and subclinical psychiatric group. A multigroup CFA compared a configural model (no equality constraints), metric model (equal factor loadings), scalar model (equal loadings and intercepts), and latent mean model (equal loadings, intercepts, and factor means). The fit indices across the first three models were similar. The configural model yielded χ^2^ = 328.181 with 86 df, RMSEA = 0.118 (95% CI: 0.10–0.13), CFI = 0.916, TLI = 0.892, and BIC = 11,348.78. Constraining factor loadings to equality (metric invariance) resulted in χ^2^ = 338.857 with 95 df, RMSEA = 0.113 (0.10–0.13), CFI = 0.915, TLI = 0.902, and BIC = 11,305.46. Adding intercept equality (scalar invariance) produced χ^2^ = 383.137 with 104 df, RMSEA = 0.115 (0.10–0.13), CFI = 0.903, TLI = 0.897, and BIC = 11,295.75. Changes in CFI and RMSEA across these three models were small (on the order of 0.01), supporting at least strong (metric + scalar) invariance. In practical terms, these findings suggest that the two groups may interpret the items in a broadly similar manner and that cautious latent-level comparisons may be possible, although the absolute fit of the models remains imperfect. The final “means equality” model, which constrained latent means to be equal, fit clearly worse (χ^2^ = 464.981 with 106 df, RMSEA = 0.130 [0.12–0.14], CFI = 0.875, TLI = 0.870, BIC = 11,365.60). This suggests that cardiological and psychiatric groups may differ in their average latent levels of meteoropathy and meteosensitivity, consistent with the observed raw-score differences. Fifth, convergent and discriminant validity were assessed. Convergent validity was tested by correlating both METEO-Q subscales with a checklist of 21 weather-related physical and psychological symptoms. In the full sample, correlations between meteoropathy and individual symptoms ranged from approximately r = 0.4 to r = 0.7, and correlations between meteosensitivity and the same symptoms were of comparable magnitudes. This pattern has been replicated in healthy adults, cardiological outpatients, and psychiatric subclinical participants. These associations suggest that higher METEO-Q scores co-occur with fatigue, irritability, headaches, sleep disturbances, cognitive difficulties, and interference with daily functioning, which matches the intended content of the scale [23,24,25]. Discriminant validity was examined using two approaches. First, the Average Variance Extracted (AVE) for Meteoropathy and Meteosensitivity ranged from 0.60 to 0.66 across subsamples, and for each factor, the AVE exceeded both the Average Shared Variance (ASV = 0.18–0.23) and the Maximum Shared Variance (MSV = 0.45–0.55). Second, the strength of the association between the METEO-Q subscales and climate-related affective constructs was modest in this study. Correlations between Meteoropathy and climate-related anxiety and climate change worry were approximately r = 0.20–0.22, and between meteosensitivity and those measures were approximately r = 0.21–0.26, whereas correlations with the symptom checklist items were much stronger. This dissociation suggests that the METEO-Q does not simply reflect general worry about global climate change or climate-related anxiety [43,44]. Instead, it primarily indexes proximal psychophysiological reactivity to ordinary atmospheric variations.

Finally, we derived preliminary percentile-based severity cut-off points to facilitate individual interpretation of the scores. For each group (healthy adults, cardiological outpatients, and psychiatric sublinical sample), the overall METEO-Q scores were divided into four provisional severity levels: low, medium, elevated, and high. These group-specific cut-off points are intended as an exploratory stratification framework for research and possible future clinical monitoring, rather than as diagnostic or triage thresholds. When these cut-off points were applied, psychiatric subclinical participants were more frequently classified into the “elevated” and “high” categories than cardiological outpatients or healthy adults. This mirrors prior observations that higher meteorological reactivity and meteorological stress exposure may be linked to clinically relevant destabilization, including emergency psychiatric presentations, involuntary admissions, suicidality, and persistent distress following severe weather exposure [35,36,37,38,42,43,44,45,47,48,49].

This study had several limitations. First, the study design was cross-sectional and relied primarily on self-reported data. No causal inference can be made regarding the direction of the effects of meteorological variations and symptom burden. In particular, although cardiological outpatients were recruited in treatment settings with clinician involvement, psychiatric status was established by self-report in a community-recruited group, and all symptom measures were self-reported by the participants. Second, the recruitment was non-probabilistic. Healthy adults and psychiatric subclinical participants were recruited using snowball sampling, whereas cardiological outpatients were recruited from treatment settings. These subgroups differed demographically; cardiological outpatients were older, and psychiatric participants were younger and predominantly female. This imbalance may have partially contributed to the group differences in the METEO-Q scores. Third, data cleaning excluded 102 observations (8.9% of all surveyed individuals) whose multivariate response patterns were considered unreliable (χ^2^ > 4.21; df = 11; *p* < 0.01), which implies that highly inconsistent profiles were removed prior to analysis. Fourth, although the two-factor model consistently outperformed the one-factor model, the absolute CFA fit indices in the Test subsample still showed RMSEA > 0.08 and CFI < 0.95. This indicates localized areas of misfit, possibly reflecting wording similarity and a small number of items per factor analyzed. Fifth, temporal stability (test–retest reliability) was not assessed in the Polish sample; therefore, it is not yet known to what extent Meteoropathy and Meteosensitivity scores reflect relatively stable traits versus more labile states. Future studies should include longitudinal designs and ecological meteorological measurements to link day-level variations in weather parameters with within-person variations in METEO-Q scores. Finally, the percentile-based severity cut-off points should be regarded as preliminary, as they have not yet been prospectively linked to objective clinical outcomes and should not be used as clinical decision thresholds until further validation.

In summary, the Polish version of the METEO-Q exhibited a replicable two-factor structure (meteoropathy and meteosensitivity) across independent subsamples of healthy adults and high internal consistency for both subscales and the total score. The results also provide preliminary evidence for approximate metric and scalar invariance across cardiological outaptient and subclinical psychiatric sample, together with higher latent severity in the subclinical sample. Convergent and discriminant validity findings were broadly consistent with the intended construct, and the preliminary percentile-based cut-off points may serve as an exploratory framework for further research. Taken together, these findings suggest that individual reactivity to ordinary weather variations can be assessed in a standardized manner; however, cross-group comparisons and possible clinical applications should be interpreted cautiously until further validation is available.

## 5. Conclusions

The present study provides the first psychometric validation of the Polish version of the Meteoropathy Questionnaire (METEO-PL) in a large mixed sample of healthy adults, cardiological outpatients, and subclinical psychiatric participants (*n* = 1128). The METEO-Q was translated and reviewed by experts and then evaluated in terms of internal consistency, factorial structure, measurement invariance, convergent and discriminant validity, and preliminary percentile-based cut-offs. The pattern of findings suggests that METEO-PL is internally consistent and structurally coherent, with preliminary support for its use in research on weather-related symptom reactivity. The broader literature cited above provides a rationale for studying these phenomena, but it does not by itself establish the clinical applicability of the present version of the instrument.

First, Polish data supported a two-factor structure of METEO-Q. In the Train subsample of healthy adults (*n* = 426), a two-factor exploratory model fit the data better than a one-factor model (χ^2^ = 67.588, df = 34, RMSEA = 0.116, CFI = 0.944, TLI = 0.909, BIC = 21.79) compared to the single-factor alternative (χ^2^ = 149.484, df = 44, RMSEA = 0.146, CFI = 0.885, TLI = 0.857, BIC = 175.02). This structure was then confirmed in an independent Test subsample (*n* = 285) using confirmatory factor analysis, where the theoretically specified two-factor model (χ^2^ = 210.192, df = 43, RMSEA = 0.120, CFI = 0.927, TLI = 0.907, BIC = 7111.00) fit better than the one-factor model (χ^2^ = 260.810, df = 44, RMSEA = 0.135, CFI = 0.906, TLI = 0.882, BIC = 7156.01). Standardized loadings were high for both latent factors: items 1–5 loaded on meteoropathy with β ranging from 0.62 to 0.84, and items 6–11 loaded on meteosensitivity with β ranging from 0.72 to 0.88. The latent correlation between Meteoropathy and Meteosensitivity was β = 0.92. We defined meteoropathy (items 1–5) as the appearance or worsening of physical and psychological symptoms in temporal association with changes in weather or atmospheric conditions. We defined meteosensitivity (items 6–11) as a biological susceptibility to meteorological changes expressed as shifts in mood, energy, cognition, and sleep [23]. In other words, meteoropathy refers to perceived symptom worsening in response to weather changes, whereas meteosensitivity refers to perceived vulnerability or reactivity to such changes. This assignment of items to factors was confirmed by the original authors [23].

Second, the internal consistency of the Polish METEO-Q was high in two independent subsamples of healthy adults. Cronbach’s α for the total score was 0.94 in both the Train subsample (*n* = 426) and the Test subsample (*n* = 285). Subscale reliability was also strong, with α = 0.86–0.89 for meteoropathy and α = 0.92–0.93 for meteosensitivity, and average item–scale correlations in the range of 0.56–0.68. Taken together, these results suggest that each factor is internally coherent and not redundant. Reliability at this level is consistent with, and in some cases numerically higher than, prior work on the original Italian METEO-Q [23] and subsequent adaptations that also recovered a two-factor solution with high internal consistency and, where assessed, acceptable short-term temporal stability [24,25]. This cross-cultural convergence suggests that subjective reactivity to weather and day-to-day atmospheric variability is a reproducible construct and not an idiosyncratic artifact of Polish respondents [23,24,25].

Third, the two-factor model demonstrated measurement invariance across clinically relevant groups. A multigroup analysis comparing cardiological outpatients and psychiatric subclinical participants supported configural, metric, and scalar (strong) invariance, with only small changes in fit indices when loadings and intercepts were constrained. This pattern suggests that both groups interpret the items in a comparable way and that scores can be cautiously compared at the latent-construct level. The additional model that constrained the latent means to equality fit clearly worse, indicating that the groups differed in their average latent severity. Consistent with this result, subclinical psychiatric participants tended to have the highest mean levels of both Meteoropathy and Meteosensitivity, followed by cardiological outpatients and healthy adults. This gradient corresponds to prior reports that psychiatric presentations, including involuntary admissions, acute destabilization of severe mental disorders, mood dysregulation, sleep disturbance, and suicidality, tend to increase under adverse or extreme meteorological conditions and thermal stress [35,36,37,38,42,45,47,48,49]. Therefore, the present data suggest that weather-related reactivity is not evenly distributed but is most pronounced in psychiatric care, where it may represent a clinically relevant dimension of the burden. The interpretation of between-group differences should still consider the demographic and recruitment differences described in the Methods section.

Fourth, convergent and discriminant validity analyses generally supported the interpretability of METEO-Q scores. Both Meteoropathy and Meteosensitivity showed moderate-to-strong correlations (approximately r = 0.40–0.70) with a checklist of 21 weather-related physical and psychological symptoms, including fatigue, irritability, headaches, sleep disturbance, cognitive difficulty, and functional interference. These associations align with the typical clinical descriptions of “feeling physically and mentally worse when the weather changes”—a pattern often referred to as “meteoropathy” in clinical and biometeorological writing [23,46]. Simultaneously, correlations between METEO-Q scores and broader climate-related affective constructs (for example, climate-change worry and climate-related anxiety) were approximately r = 0.20–0.26, and the Average Variance Extracted for each factor exceeded the shared variance with those climate-related measures. This pattern suggests that the METEO-Q does not simply reflect global climate-related concerns, which are often described as anticipatory, existential, and future-oriented distress [43,44], but instead primarily captures proximal psychophysiological reactivity to ordinary weather variations in day-to-day life [23,24,25].

Finally, the present study derived preliminary percentile-based cut-offs defining four severity cut-off points (low, medium, risk, and high) separately for healthy adults, cardiological outpatients, and the subclinical psychiatric group. Because these thresholds are group-specific, they should be treated as an exploratory framework for research rather than as evidence of established clinical stratification. Subclinical psychiatric individuals were more often classified in the “risk” and “high” ranges than cardiological outpatients or healthy adults. These group differences should, however, be interpreted cautiously, because the thresholds are preliminary and the studied subsamples differed in demographic composition and recruitment strategy. Several constraints were applied. All data were cross-sectional and based on self-reports, preventing causal inference regarding the directionality between meteorological variation and symptom severity. Recruitment was non-probabilistic, and the clinical subsamples differed demographically from the healthy adult sample in terms of age and sex. Temporal stability (test–retest reliability) was not assessed in the Polish version, unlike other adaptations, which reported acceptable short-term stability [25]. Finally, although the two-factor confirmatory model consistently outperformed the one-factor alternative, absolute fit indices still indicated localized misfit, which likely reflects the short length of each subscale and the partial overlap between items. These limitations indicate that future studies should include longitudinal designs, explicit test–retest data, and the linkage of within-person METEO-Q variation to objective daily level meteorological parameters. Such work is clinically relevant because extreme weather exposure and broader climatic instability have been associated with persistent anxiety, insomnia, depressive symptoms, and stress-related symptoms after events such as floods and wildfires [43,44]. Day-to-day meteorological stress also appears to contribute to cardiometabolic strain [34,39,40,41] and psychiatric destabilization in vulnerable groups [35,45].

In summary, the Polish METEO-Q demonstrated a replicable two-factor structure, high internal consistency, and validity findings that were broadly consistent with the intended construct. The results also provided preliminary support for approximate cross-group comparability at the latent level and indicated higher latent severity in the subclinical psychiatric sample. In addition, the proposed group-specific cut-off points may be useful as an exploratory framework for future research. Overall, the Polish METEO-Q appears suitable for research use, whereas stronger claims regarding cross-group comparability, clinical validity, and clinical applicability require further validation.

## Figures and Tables

**Table 1:** Sample characteristics.

Characteristic	Healthy Adults *n* = 711 ^1^	Cardiological *n* = 194 ^1^	Psychiatric *n* = 223 ^1^	*p*
Sex				
Male	267 (37.5%)	101 (52%)	47 (21%)	
Female	444 (62.5%)	93 (48%)	176 (79%)	
Age (in years)	40.0 (20.8), 18.0–89.0	64.5 (14.1), 18.0–89.0	25.0 (5.9), 19.0–72.0	
Education Level				<0.001
Primary	18 (2.7%)	19 (13%)	4 (1.9%)	
Lower Secondary	7 (1.1%)	7 (4.8%)	3 (1.4%)	
Professional	0 (0%)	0 (0%)	0 (0%)	
Secondary	237 (36%)	84 (58%)	100 (46%)	
Post Secondary	52 (7.9%)	10 (6.8%)	15 (6.9%)	
Higher	341 (52%)	26 (18%)	94 (44%)	

^1^ *n* (%); Median (mad), Min–Max.

**Table 2:** Characteristics of the Train and Test sample.

Variable	Train *n* = 426 ^1^	Test *n* = 285 ^1^	*p* ^2^
Sex			0.378
Male	159 (37.4%)	108 (37.9%)	
Female	267 (62.6%)	177 (62.1%)	
Age (in years)	42.0 (20.8), 18.0–89.0	38.0 (20.8), 18.0–85.0	0.241
Education Level			0.947
Primary	10 (2.6%)	8 (3.0%)	
Lower Secondary	5 (1.3%)	2 (0.7%)	
Professional	0 (0%)	0 (0%)	
Secondary	138 (36%)	99 (37%)	
Post Secondary	32 (8.3%)	20 (7.4%)	
Higher	201 (52%)	140 (52%)	

^1^ *n* (%); Median (mad), Min–Max; ^2^ Welch Two Sample *t*-test; Pearson’s Chi-squared test. Note: *p*-value χ^2^ of independence test.

**Table 3:** Summary of Cronbach’s alpha reliability of METEO-Q scores in the train sample (*n* = 426).

Sample	Scale	Cronbach’s α	95% Confidence	Average r	M (SD)
Train					
	Total score	0.94	0.93–0.95	0.59	14.15 (9.65)
	Meteoropathy	0.86	0.84–0.88	0.56	7.21 (4.52)
	Meteosensitivity	0.93	0.91–0.94	0.68	6.94 (5.64)
Test					
	Total score	0.94	0.93–0.95	0.60	14.46 (9.77)
	Meteoropathy	0.89	0.87–0.91	0.61	7.34 (4.7)
	Meteosensitivity	0.92	0.9–0.93	0.64	7.12 (5.51)

**Table 4:** Descriptive statistics for the METEO-Q in Polish sample.

Sample	Scale	Min–Max	M (SD)	Me (Mad)	Skew	Kurt	W
Healthy Adults							
	Meteoropathy	0–20	7.24 (4.61)	7 (5.9)	0.19	−0.70	0.97
	Meteosensitivity	0–24	7.03 (5.62)	6 (5.9)	0.59	−0.43	0.94
Cardiological							
	Meteoropathy	0–20	6.13 (4.36)	6 (4.4)	0.57	−0.22	0.96
	Meteosensitivity	0–24	7.34 (5.84)	7 (5.9)	0.57	−0.30	0.94
Psychiatric							
	Meteoropathy	0–20	9.58 (4.26)	9 (4.4)	−0.07	−0.55	0.98
	Meteosensitivity	0–23	8.97 (5.58)	9 (7.4)	0.31	−0.78	0.97

**Table 5:** Summary of fit indices for one and two dimensional approaches to METEO-Q in the Train sample (*n* = 426).

Dimmensions	χ^2^	df	RMSEA	95% Confidence	TLI	CFI	BIC	η^2^
1	149.484	44	0.146	0.13–0.16	0.857	0.885	175.02	0.594
2	67.588	34	0.116	0.1–0.13	0.909	0.944	21.79	0.643

**Table 6:** Summary of exploratory factor analysis in the Train sample (*n* = 426).

Item	Total Score	MR1	MR2
1	0.53	0.38	0.18
2	0.72	0.73	0.02
3	0.78	0.91	−0.09
4	0.78	0.79	0.03
5	0.77	0.77	0.03
6	0.84	0.67	0.21
7	0.81	0.39	0.46
8	0.79	0.26	0.56
9	0.76	0.03	0.78
10	0.81	−0.08	0.95
11	0.84	0.08	0.82

**Table 7:** CFA model fit indices for unidimensional, 2-dimensional and empirical models.

Model	χ^2^	df	RMSEA	95% Confidence	TLI	CFI	BIC
Unidimensional	260,810	44	0.135	0.12–0.15	0.882	0.906	7156.01
2-dimensional	210,192	43	0.120	0.1–0.14	0.907	0.927	7111.00
Empirical	224,436	43	0.125	0.11–0.14	0.899	0.921	7125.24

**Table 8:** Path coefficients of confirmatory factor analysis.

		b	95% Confidence	β	Z
Meteoropathy					
	Item 1	1.00		0.62	
	Item 2	1.57	1.3–1.85	0.84	11.14
	Item 3	1.54	1.27–1.81	0.84	11.11
	Item 4	1.54	1.26–1.82	0.81	10.82
	Item 5	1.54	1.27–1.82	0.83	11.02
Meteosensitivity					
	Item 6	1.00		0.83	
	Item 7	0.92	0.8–1.03	0.79	15.41
	Item 8	0.86	0.74–0.97	0.78	15.15
	Item 9	0.93	0.8–1.07	0.72	13.67
	Item 10	1.01	0.89–1.13	0.83	16.70
	Item 11	1.08	0.96–1.19	0.88	18.29
Meteoropathy					
	Meteosensitivity	0.52	0.39–0.65	0.92	7.83

**Table 9:** Summary of measurement invariance due to type of disease.

Invariance	χ^2^	df	*p*	RMSEA	95% Confidence	TLI	CFI	BIC
Configural	328.181	86		0.118	0.1–0.13	0.892	0.916	11,348.78
Metric	338.857	95	0.299	0.113	0.1–0.13	0.902	0.915	11,305.46
Scalar	383.137	104	<0.001	0.115	0.1–0.13	0.897	0.903	11,295.75
Means	464.981	106	<0.001	0.130	0.12–0.14	0.870	0.875	11,365.60

**Table 10:** Summary of discriminant analysis using HTMT method.

Estimator	Healthy Adults		Subclinical Adults	
	meteoropathy	meteosensitivity	meteoropathy	meteosensitivity
Cronbach α	0.88	0.92	0.87	0.90
McDonald’s Ω	0.88	0.92	0.88	0.90
AVE	0.61	0.66	0.60	0.61
ASV	0.20	0.23	0.18	0.21
MSV	0.52	0.55	0.45	0.46

**Table 11:** Summary of correlations between meteoropathy and meteosensitivity, Climate Anxiety, Climate Change Worries and Symptoms.

Correlates	Meteoropathy	Meteosensitivity
Wahania nastroju	0.69 (0.66; 0.72)	0.67 (0.63; 0.7)
Duża wrażliwość na wydarzenia zewnętrzne	0.62 (0.58; 0.65)	0.64 (0.61; 0.68)
Przewlekłe zmęczenie	0.61 (0.57; 0.64)	0.56 (0.52; 0.6)
Drażliwość, nerwowość	0.58 (0.54; 0.62)	0.55 (0.51; 0.59)
Nudności	0.58 (0.54; 0.61)	0.52 (0.48; 0.56)
Nieokreślone poczucie dyskomfortu	0.56 (0.51; 0.6)	0.58 (0.54; 0.62)
Apatia, brak radości	0.57 (0.52; 0.6)	0.53 (0.49; 0.57)
Problemy z koncentracją	0.51 (0.47; 0.56)	0.5 (0.45; 0.54)
Nieokreślone bóle, bóle stawów, bóle mięśni	0.39 (0.34; 0.44)	0.47 (0.42; 0.52)
Bóle głowy	0.43 (0.38; 0.48)	0.46 (0.41; 0.5)
Nadmierna senność	0.44 (0.39; 0.49)	0.41 (0.36; 0.46)
Zawroty głowy	0.39 (0.34; 0.44)	0.44 (0.39; 0.48)
Depresja	0.43 (0.38; 0.48)	0.42 (0.37; 0.47)
Nadmierny apetyt	0.41 (0.35; 0.45)	0.42 (0.37; 0.47)
Lęk	0.39 (0.34; 0.44)	0.41 (0.36; 0.46)
Zmiany w libido	0.38 (0.33; 0.43)	0.4 (0.35; 0.45)
Zaburzenia rytmu serca	0.29 (0.23; 0.34)	0.39 (0.33; 0.44)
Nudności	0.29 (0.24; 0.35)	0.38 (0.32; 0.43)
Bezsenność	0.34 (0.29; 0.39)	0.38 (0.33; 0.43)
Brak apetytu	0.31 (0.26; 0.37)	0.36 (0.3; 0.41)
Problemy trawienne	0.29 (0.24; 0.34)	0.36 (0.31; 0.41)
Climate Change Worry	0.22 (0.16; 0.28)	0.28 (0.22; 0.33)

**Table 12:** Summary of correlations between meteoropathy and meteosensitivity, Climate Anxiety, Climat Change Worries and Symptoms in tested Samples.

Sample	Correlates	Meteoropathy	Meteosensitivity
Healthy Adults			
	Wahania nastroju	0.69 (0.65; 0.73)	0.68 (0.64; 0.72)
	Duża wrażliwość na wydarzenia zewnętrzne	0.63 (0.59; 0.68)	0.65 (0.61; 0.69)
	Przewlekłe zmęczenie	0.61 (0.56; 0.66)	0.55 (0.5; 0.6)
	Nieokreślone poczucie dyskomfortu	0.56 (0.5; 0.61)	0.61 (0.56; 0.66)
	Drażliwość, nerwowość	0.6 (0.55; 0.65)	0.58 (0.53; 0.63)
	Apatia, brak radości	0.58 (0.53; 0.63)	0.55 (0.5; 0.6)
	Ospałość i zmęczenie w pracy lub podczas codziennych czynności	0.58 (0.53; 0.63)	0.52 (0.46; 0.57)
	Problemy z koncentracją	0.53 (0.47; 0.58)	0.53 (0.47; 0.58)
	Nieokreślone bóle, bóle stawów, bóle mięśni	0.46 (0.4; 0.52)	0.52 (0.47; 0.57)
	Bóle głowy	0.45 (0.39; 0.51)	0.47 (0.41; 0.52)
	Nadmierna senność	0.46 (0.4; 0.51)	0.41 (0.35; 0.47)
	Zawroty głowy	0.41 (0.34; 0.47)	0.46 (0.4; 0.52)
	Zmiany w libido	0.45 (0.38; 0.5)	0.46 (0.4; 0.51)
	Zaburzenia rytmu serca	0.35 (0.29; 0.42)	0.44 (0.37; 0.5)
	Nadmierny apetyt	0.37 (0.3; 0.43)	0.42 (0.35; 0.48)
	Depresja	0.37 (0.3; 0.43)	0.4 (0.33; 0.46)
	Lęk	0.37 (0.31; 0.43)	0.4 (0.33; 0.46)
	Nudności	0.33 (0.27; 0.4)	0.39 (0.33; 0.45)
	Bezsenność	0.37 (0.31; 0.44)	0.39 (0.32; 0.45)
	Problemy trawienne	0.31 (0.24; 0.38)	0.39 (0.32; 0.45)
	Brak apetytu	0.33 (0.26; 0.39)	0.38 (0.31; 0.44)
	Climate Change Worry	0.23 (0.16; 0.3)	0.28 (0.21; 0.35)
	Climate Change Anxiety	0.16 (0.09; 0.23)	0.21 (0.14; 0.28)
Cardiological			
	Wahania nastroju	0.72 (0.64; 0.78)	0.62 (0.52; 0.7)
	Duża wrażliwość na wydarzenia zewnętrzne	0.57 (0.47; 0.66)	0.54 (0.43; 0.63)
	Przewlekłe zmęczenie	0.56 (0.45; 0.65)	0.53 (0.42; 0.63)
	Nieokreślone poczucie dyskomfortu	0.54 (0.43; 0.64)	0.45 (0.33; 0.56)
	Zawroty głowy	0.51 (0.4; 0.61)	0.43 (0.3; 0.53)
	Ospałość i zmęczenie w pracy lub podczas codziennych czynności	0.49 (0.38; 0.59)	0.5 (0.38; 0.6)
	Apatia, brak radości	0.49 (0.38; 0.59)	0.41 (0.29; 0.52)
	Problemy z koncentracją	0.47 (0.36; 0.58)	0.38 (0.25; 0.5)
	Zaburzenia rytmu serca	0.44 (0.32; 0.55)	0.38 (0.25; 0.5)
	Zmiany w libido	0.42 (0.29; 0.53)	0.34 (0.21; 0.46)
	Nadmierny apetyt	0.41 (0.28; 0.52)	0.34 (0.21; 0.46)
	Depresja	0.4 (0.28; 0.51)	0.33 (0.2; 0.45)
	Drażliwość, nerwowość	0.4 (0.28; 0.52)	0.35 (0.22; 0.47)
	Problemy trawienne	0.4 (0.28; 0.51)	0.35 (0.22; 0.47)
	Nieokreślone bóle, bóle stawów, bóle mięśni	0.39 (0.26; 0.5)	0.36 (0.23; 0.48)
	Bóle głowy	0.34 (0.21; 0.46)	0.37 (0.24; 0.49)
	Lęk	0.35 (0.22; 0.47)	0.33 (0.2; 0.45)
	Brak apetytu	0.35 (0.22; 0.47)	0.3 (0.16; 0.42)
	Climate Change Anxiety	0.32 (0.19; 0.44)	0.35 (0.21; 0.46)
	Nudności	0.33 (0.2; 0.45)	0.31 (0.18; 0.43)
	Climate Change Worry	0.29 (0.15; 0.41)	0.31 (0.18; 0.44)
	Bezsenność	0.28 (0.15; 0.41)	0.31 (0.18; 0.43)
	Nadmierna senność	0.28 (0.14; 0.4)	0.31 (0.17; 0.43)
Psychiatric			
	Duża wrażliwość na wydarzenia zewnętrzne	0.57 (0.48; 0.66)	0.69 (0.61; 0.75)
	Wahania nastroju	0.6 (0.51; 0.68)	0.65 (0.57; 0.72)
	Drażliwość, nerwowość	0.58 (0.48; 0.66)	0.59 (0.49; 0.67)
	Przewlekłe zmęczenie	0.53 (0.43; 0.62)	0.56 (0.46; 0.65)
	Nieokreślone poczucie dyskomfortu	0.5 (0.39; 0.59)	0.55 (0.45; 0.64)
	Apatia, brak radości	0.51 (0.41; 0.6)	0.51 (0.41; 0.61)
	Ospałość i zmęczenie w pracy lub podczas codziennych czynności	0.5 (0.39; 0.59)	0.5 (0.39; 0.59)
	Depresja	0.44 (0.33; 0.54)	0.48 (0.37; 0.58)
	Lęk	0.37 (0.25; 0.48)	0.46 (0.35; 0.56)
	Nadmierny apetyt	0.45 (0.33; 0.55)	0.46 (0.35; 0.56)
	Problemy z koncentracją	0.4 (0.28; 0.51)	0.45 (0.34; 0.55)
	Bóle głowy	0.33 (0.21; 0.45)	0.44 (0.32; 0.54)
	Nadmierna senność	0.38 (0.26; 0.49)	0.44 (0.32; 0.54)
	Nieokreślone bóle, bóle stawów, bóle mięśni	0.26 (0.13; 0.38)	0.42 (0.31; 0.53)
	Nudności	0.22 (0.09; 0.34)	0.4 (0.28; 0.51)
	Bezsenność	0.28 (0.16; 0.4)	0.4 (0.28; 0.5)
	Zawroty głowy	0.23 (0.1; 0.35)	0.36 (0.23; 0.47)
	Zaburzenia rytmu serca	0.16 (0.02; 0.28)	0.32 (0.19; 0.44)
	Brak apetytu	0.19 (0.06; 0.32)	0.32 (0.19; 0.43)
	Problemy trawienne	0.23 (0.1; 0.35)	0.31 (0.18; 0.42)
	Zmiany w libido	0.2 (0.07; 0.32)	0.28 (0.15; 0.4)
	Climate Change Anxiety	0.2 (0.07; 0.32)	0.26 (0.13; 0.38)
	Climate Change Worry	0.22 (0.08; 0.34)	0.21 (0.08; 0.34)

## Data Availability

Data available upon request.

## Supplementary Materials

The following supporting information can be downloaded at: https://www.mdpi.com/article/10.3390/jcm15082853/s1, Supplementary Table S1. Cut-off points for individual diagnosis; Supplementary Table S2. Frequencies in different levels of meteoropathy and meteosensitivity.
