# Supplementary material for: Polish Adaptation and Psychometric Validation of the METEO-Q in Healthy, Cardiac, and Psychiatric Samples
Source: J Clin Med. 2026 Apr 9;15(8):2853. doi: 10.3390/jcm15082853 (PMC13116603; doi:10.3390/jcm15082853)
Supplement: Supplementary file 1 [file jcm-15-02853-s001.zip › Supplementary Table S1.pdf]

Supplementary Table S1. Cut-off points for individual diagnosis

Sample	Scale	Min-Max	M (SD)	Low	Medium	Risk	High
Healthy Adults							
	Meteoropatia	0 - 20	7.24 (4.63)	< 4	< 7	< 11	< 20
	Meteosensytywność	0 - 24	6.95 (5.6)	< 2	< 6	< 11	< 24
Cardiological							
	Meteoropatia	0 - 20	6.13 (4.36)	< 3	< 6	< 9	< 20
	Meteosensytywność	0 - 27	7.33 (5.85)	< 2	< 7	< 11	< 27
Psychiatric							
	Meteoropatia	0 - 20	9.58 (4.3)	< 6.2	< 9	< 13	< 20
	Meteosensytywność	0 - 23	9.03 (5.57)	< 4	< 9	< 12.8	< 23
