# Supplementary material for: Polish Adaptation and Psychometric Validation of the METEO-Q in Healthy, Cardiac, and Psychiatric Samples
Source: J Clin Med. 2026 Apr 9;15(8):2853. doi: 10.3390/jcm15082853 (PMC13116603; doi:10.3390/jcm15082853)
Supplement: Supplementary file 1 [file jcm-15-02853-s001.zip › Supplementary Table S2.pdf]

Supplementary Table S2. Frequencies in different levels of meteoropathy and meteosensitivity

Sample	Scale	Level (score)			
		Low	Medium	Risk	High
Healthy Adults					
	Meteoropathy	205 (29.5%)	159 (22.9%)	191 (27.5%)	139 (20%)
	Meteosensitivity	183 (26.6%)	183 (26.6%)	156 (22.7%)	166 (24.1%)
Cardiological					
	Meteoropathy	61 (31.6%)	48 (24.9%)	44 (22.8%)	40 (20.7%)
	Meteosensitivity	49 (25.3%)	57 (29.4%)	41 (21.1%)	47 (24.2%)
Psychiatric					
	Meteoropathy	55 (25.1%)	58 (26.5%)	61 (27.9%)	45 (20.5%)
	Meteosensitivity	56 (26.3%)	68 (31.9%)	36 (16.9%)	53 (24.9%)
